# Supplementary material for: elPrep 4: A multithreaded framework for sequence analysis
Source: PLoS One. 2019 Feb 13;14(2):e0209523. doi: 10.1371/journal.pone.0209523 (PMC6373927; doi:10.1371/journal.pone.0209523)
Supplement: S1 Appendix — We describe how to express the optical duplicate marking algorithm from Picard/GATK 4 as a parallel, single-pass algorithm in the new elPrep 4 framework. (PDF) [file pone.0209523.s001.pdf]

S1 Appendix: Optical duplicate marking in elPrep 4

Charlotte Herzeel[1◑*], Pascal Costanza[1◑], Dries Decap[1,2], Jan Fostier[1,2], Wilfried Verachtert[1]

1 ExaScience Life Lab, imec, Leuven, Belgium
2 Department of Information Technology, Ghent University - imec, Ghent, Belgium

◑These authors contributed equally to this work.
* Charlotte.Herzeel@imec.be

Expressing optical duplicate marking in elPrep 4

There are two reasons why read duplicates may occur in a BAM file [1]. Firstly, the same DNA fragment may be sequenced multiple times during the sample preparation step because of how library construction using PCR works. This creates *PCR duplicates*. Secondly, *optical duplicates* may occur because of mistakes made by the sequencer, namely when a single amplification cluster is incorrectly identified as multiple clusters by the optical sensor. In both cases, this will result in multiple reads being recorded that may look similar in the BAM file, but are not necessarily identical.

In general, it is desirable to mark duplicates before statistical analysis of a BAM file as read duplicates are not independent observations. The Picard/GATK 4 duplicate marking tool also distinguishes between optical and PCR duplicates to generate a metrics file that specifies the ratio of optical versus PCR duplicates. This reveals useful information about the quality of the sample and sequencing run. We previously described a parallel algorithm we derived for elPrep from the Picard algorithm for duplicate marking [2]. In elPrep 4, we extend it to include optical duplicate marking.

Picard/GATK 4 algorithm

The Picard/GATK 4[1] strategy for identifying duplicates is to closely compare reads that map to the exact same location in the reference genome. Optical duplicates are then distinguished from PCR duplicates by inspecting the tile coordinates that are recorded for each read (see QNAME in the SAM record [3]). If two reads are closely located on the same tile, they are likely to be optical duplicates.

The Picard/GATK 4 algorithm for duplicate marking is a sequential, multi-pass algorithm that reads the input file multiple times. It consists of the following phases:

1. Sort the reads according to mapping coordinates and keep track of the original file positions of the reads.

2. Identify groups of potential duplicates within the sorted list by grouping together all reads mapping to the same position. For each of these groups, identify the read with the highest quality score, still keeping track of the file positions of all other reads in that group. The latter are considered duplicates of the former.

[1]GATK 4 merges the GATK and Picard softwares.

3. Identify optical duplicates in these groups of duplicates. For this, do a pairwise comparison between all reads in each group. Calculate the distance of the reads using their tile coordinates, and if this distance is below a certain threshold, count one of them as an optical duplicate. Preference is given to the non-duplicate read of a group as the origin of optical duplicates. Listing 1 shows simplified pseudo code for this phase.

4. Write a new output file by copying the reads from the original file, but use the file positions identified in step 2 to identify which reads are marked as a duplicate. Based on the counts in step 3, generate a metrics file that, among others, contains the ratio of PCR/optical duplicates.

Each of these phases implement nested loops that iterate sequentially over the full read data. In contrast, the algorithms for duplicate marking in elPrep are parallelized.

```
func close(read1, read2, threshold){
    return |read1.TileX - read2.TileX| <= threshold &&
           |read1.TileY - read2.TileY| <= threshold
}

func markOpticalDuplicates(dups, threshold){
    ctr := 0
    for(i := 0; i < len(dups); i++):
        read1 := dups[i]
        for(j := i + 1;  j < len(dups); j++):
            read2 := dups[j]
            if read2.optical:
                continue
            if read1.Tile != read2.Tile:
                continue
            if close(read1, read2, threshold):
                read2.optical = true
                ctr++
     return ctr
}
```

Listing 1. Pseudo code for optical duplicate marking within a list of duplicates in Picard/GATK 4 (simplified).

A parallel optical duplicate marking algorithm in elPrep 4

The elPrep 4 algorithm for optical duplicate marking produces the same output as the algorithm from Picard/GATK 4, yet it has a very different structure. In previous work, we discussed how the Picard algorithm for duplicate marking can be reformulated as a filter [2]. This allows elPrep to perform duplicate marking using only a single pass through the read data compared to the multi-pass algorithm in Picard. This removes considerable I/O overhead and allows elPrep to parallelize the execution of duplicate marking, which greatly speeds up the runtime of duplicate marking [2].

We cannot add optical duplicate marking as a filter operation. In the previous sections, we explained that the general duplicate marking algorithm identifies duplicates based on mapping positions, without distinguishing between optical or PCR duplicates. Optical duplicates can be identified among those duplicates by comparing their respective distances based on their tile coordinates. For this to work, the general duplicate marking must already be done for all reads.

We therefore implement optical duplicate marking as an operation on the whole read set. This is separate from the general duplicate marking filter in elPrep. Our algorithm for optical duplicate marking consists of two phases:

1. When the duplicate marking filter is executed, the reads that have duplicates are identified and stored in a table that we can reuse for the optical duplicate marking. As a first step, we iterate over all the reads –stored in memory– and identify all reads that are tagged as duplicates. For these reads, we look up the *origin*, the read for which they are duplicates, in the table created by the general duplicates marking filter. We compute the distance between duplicate and origin to determine if the read is a duplicate of the origin, and if so, increment our count. We also store for each origin what its duplicates are.

2. For each origin read we next do a pairwise comparison among its duplicates identified in step 1.

These phases are implemented as parallel algorithms. Simplified pseudo code is shown in Listing 2. The first phase is implemented as a parallel range-reduce pattern. The range-reduce has a target data structure (reads) and two functions, a *map* and a *reduce*[2], as arguments. The idea of the range-reduce is that it implements a parallel loop that splits up the iterations over the reads into parallel tasks.

The map function checks if a read is a duplicate, in which case it will look up the origin, which is the read that caused it to be marked as a duplicate in the first place. It stores the read with the origin, and calculates the distance to that origin to decide whether to count it as an optical duplicate or not. The storing of the read with the origin needs to be synchronized as different parallel tasks may find the same origin for the reads they are processing. We implement this using atomic compare-and-swap operations [7] (not shown).

The reduce function is there to combine the results of the parallel tasks instantiated by the map function. It describes how to combine the results of two tasks. In this case, this is done by adding up the optical duplicate counts of the map tasks. The actual implementation of the parallel range-reduce takes care of creating reduction tasks for combining the results of the map tasks while minimizing synchronization.

The second phase of the algorithm is also implemented as a parallel range-reduce, but this one iterates over the duplicate origins. The map function defines what needs to be done for a single origin. It executes a loop that does a pairwise comparison of its duplicates to count the number of optical duplicates. The reduce function again combines the counts from the map tasks. Finally, the counts from phase 1 and phase 2 are combined to create the total optical duplicate count.

The pseudo code we discussed here, both for the Picard/GATK 4 and elPrep 4 algorithms, gives a good idea of the general structure of their implementations, but it is still a stark simplification of the actual code. It omits numerous details, for example about other statistics that are calculated, or specific orderings that are enforced on the reads before optical marking, and so on. All in all, the elPrep 4 implementation of optical duplicate marking is around 650 lines of Go code versus a few thousand lines of Java code in Picard/GATK 4.

[2]This form of map/reduce is different from Hadoop-style MapReduce [4], but has a long tradition in functional programming [5] and is known to have efficient parallel implementations [6].

```
func markOpticalDuplicates(reads, origins, threshold) {
    // phase 1
    result1 := parallel.Reduce(
        reads,
        // map
        func lambda(read) {
            if duplicate(read) :
                origin = origins[read]
                origin.addDup(read)
                if close(origin, read, threshold) :
                    return 1
            return 0
        },
        // reduction
        func lambda(ctr1, ctr2) {
            return ctr1 + ctr2
        }
    )
    // phase 2
    result2 := parallel.Reduce(
        origins,
        // map
        func lambda(origin) {
            dups := origin.getDups()
            ctr := 0
            for i := 0; i < len(dups); i++ {
                read1 := dups[i]
                for j := i+1; j < len(dups); j++ {
                    read2 := dups[j]
                    if read2.optical :
                        continue
                    if read1.Tile != read2.Tile :
                        continue
                    if close(read1, read2, threshold) :
                        read2.optical = true
                        ctr++
                }
            }
            return ctr
        },
        // reduction
        func lambda(ctr1, ctr2) {
            return ctr1 + ctr2
        }
    )
    return result1 + result2
}
```

Listing 2. Pseudo code for parallelized optical duplicate marking elPrep 4 (simplified).

References

1. Wingett S. Illumina Patterned Flow Cells Generate Duplicated Sequences; 2017. Available from: `https://sequencing.qcfail.com/articles/illumina-patterned-flow-cells-generate-duplicated-sequences/` [cited September 26, 2018].

2. Herzeel C, Costanza P, Decap D, Fostier J, Reumers J. elPrep: High-Performance Preparation of Sequence Alignment/Map Files for Variant Calling. PLoS ONE. 2015;10(7). doi:10.137/journal.pone.0138868.

3. Li H, Hansaker B, Wysoker A, Fennell T, Ruan J, Homer N, et al. The Sequence Alignment/Map format and SAMtools. Bioinformatics. 2009;25(16):2078–2079. doi:10.1093/bioinformatics/btp352.

4. Dean J, Ghemawat S. MapReduce: A Flexible Data Processing Tool. Communications of the ACM. 2010;53(1):72–77. doi:10.1145/1629175.1629198.

5. Abelson H, Sussman GJ, Sussman J. Structure and Interpretation of Computer Programs. Cambridge, Massachusetts: The MIT Press; 1996.

6. Blelloch G. NESL. In: Padua D, editor. Encyclopedia of Parallel Computing. Boston, Massachusetts: Springer; 2011.

7. McCool M, Robison AD, Reinders J. Structured Parallel Programming - Patterns for Efficient Computation. Waltham, Massachusetts: Morgan Kaufman; 2012.
