# Supplementary material for: elPrep 4: A multithreaded framework for sequence analysis
Source: PLoS One. 2019 Feb 13;14(2):e0209523. doi: 10.1371/journal.pone.0209523 (PMC6373927; doi:10.1371/journal.pone.0209523)
Supplement: S2 Appendix — We explain how to express the base quality score recalibration and application algorithms (BQSR) from GATK 4 as a parallel, map-reduce algorithm in the new elPrep 4 framework. (PDF) [file pone.0209523.s002.pdf]

S2 Appendix: Expressing base quality score recalibration (BQSR) in elPrep 4

Charlotte Herzeel[1◑*], Pascal Costanza[1◑], Dries Decap[1,2], Jan Fostier[1,2], Wilfried Verachtert[1]

1 ExaScience Life Lab, imec, Leuven, Belgium
2 Department of Information Technology, Ghent University - imec, Ghent, Belgium

◑These authors contributed equally to this work.
* Charlotte.Herzeel@imec.be

Expressing BQSR in elPrep 4

The purpose of base quality score recalibration is to correct for biases in the quality scores assigned by the sequencer. The idea is to compute new base quality scores for each read by applying an error model derived from the full data set. Specifically, an empirical error model is computed that takes into account how error values vary in terms of base call features such as the cycle at which a base was sequenced, the context or bases sequenced before the base, and so on. It also assumes that all non-reference bases in a read that do not match a known variant site in a dbSNP file, are sequencing errors. This specialised error model is then used in a Bayesian statistical model that for each base call computes a new base quality score by checking the expected quality score from the sequencer against the recorded observations and errors for that type of base call.

GATK algorithm

Base quality score recalibration (BQSR) is implemented using two separate tools in GATK 4, namely BaseRecalibrator and ApplyBQSR. The BaseRecalibrator tool is used to compute a recalibration file that contains empirical information about the base quality scores in a BAM file and for what types of bases they occur. The recalibration file can be loaded by the ApplyBQSR tool that uses the information to instantiate a hierarchical Bayesian statistical model to compute a new base quality score for each base in every read in the BAM file.

Concretely, the algorithm for BaseRecalibrator looks as follows:

1. Iterate over all reads in the BAM file and compute three tables based on the base quality scores reported by the sequencer:
   (a) Table 1: $(readgroup \times base\ quality \rightarrow observations, errors, empirical\ quality)$.
   This table maps each read group combined with each base quality score reported by the sequencer onto the *observations*, the number of times a particular combination of a read group and a base quality score occurs in the BAM file, and *errors*, the number of times that a base with this quality score is considered a mismatch by the aligner compared to the reference genome. The empircal quality is calculated with a Bayesian estimator from the observations and errors, using the base quality as a prior.

(b) Table 2: $(\mathit{readgroup} \times \mathit{base\ quality} \times \mathit{cycle} \rightarrow \mathit{observations}, \mathit{errors}, \mathit{empirical\ quality})$.
This table is similar to Table 1 except that it is more specialised. The *cycle* in the key indicates the cycle at which the base was sequenced. The idea is that DNA sequenced in later cycles produces less reliable bases and qualities.

(c) Table 3: $(\mathit{readgroup} \times \mathit{base\ quality} \times \mathit{context} \rightarrow \mathit{observations}, \mathit{errors}, \mathit{empirical\ quality})$.
This table is also similar to Table 1 except that it is also more specialised. The *context* in the key is the type of base sequenced before the base for which the observations and errors are recorded.

2. From the above tables, derive the following tables:

   (a) Table 4: a quantized quality score table that is a remapping of quality scores onto quantized scores, a regrouping of scores via hierarchical clustering.

   (b) Table 5: $(\mathit{read\ group} \rightarrow \mathit{observations}, \mathit{errors}, \mathit{empirical\ quality})$
   This table computes per read group: the total number of observations, total number of errors, the average quality score, and the empirical quality score calculated using a Bayesian estimator from these totals.

3. Write out these tables as a recalibration file (.recal).

The algorithm for the ApplyBQSR tool roughly looks as the simplified pseudo code in Listing 1. It is a loop that iterates over all reads in a BAM file, and for all bases of all reads calculates a new base quality score. This base quality score is calculated using a hierarchical Bayesian estimator that uses the observations and errors from Table 1, 2 and 3, and uses the average base quality score from Table 5 as a prior.

```
func ApplyBQSR(reads, recal) {
    tab1, tab2, tab3, tab4, tab5 := parseRecalFile(recal)
    for read in reads {
        for i := 0; i < len(read); i++ {
            qual := read.getBaseQual(i)
            rg := read.getReadGroup()
            cycle := read.getCycle(i)
            context := read.getContext(i)
            s1 := tab1[rg,qual]
            s2 := tab2[rg,qual,cycle]
            s3 := tab3[rg,qual,context]
            prior := tab5[rg]
            est := hierarchicalBayesianEstimate(prior,s1,s2,s3)
            read.Qual[i] = tab4[est]
        }
    }
}
```

Listing 1. Pseudo code for ApplyBQSR in GATK 4 (simplified).

The GATK 4 code for these algorithms parses the reads two times, once for the BaseRecalibrator tool, and once for the ApplyBQSR tool. It also leaves data as much as possible on disk so that they need to be repeatedly reloaded. For example, the reference FASTA file, which is needed for counting the errors for the tables in the BaseRecalibrator tool, is manipulated on disk. Also, none of the code is parallelized.

A parallel BQSR algorithm in elPrep 4

The basic structures of the elPrep 4 algorithms for BQSR are similar to the GATK 4 algorithms, except that they are parallelized and reformulated to fit into the elPrep framework. The recalibration step is implemented as a parallel algorithm that operates on the whole set of reads. It cannot be formulated as a filter because the BQSR algorithm from GATK 4 –which we want to reproduce in terms of outcome– expects a number of processing steps such as duplicate marking to already be applied.

Simplified pseudo code for the calculation of recalibration tables is shown in Listing 2. The arguments to the BaseRecalibrator are the reads, a FASTA file for the reference genome, and a VCF file that contains known variant sites, which are to be excluded from BQSR counts (like dbsnp). First, the reference FASTA file is loaded. We have implemented this using *memory-mapped files* [1], which allows the FASTA file to be treated as if it were already in main memory. To make this work, we designed our own *elfasta* format, which makes it more convenient to access the data as a memory-mapped file.

The bulk of the algorithm is a parallel range-reduce that iterates over every read, computes properties such as the cycle and context of each base in that read (in the form of arrays), and finally executes a loop that for every base of the read fills in the recalibration tables using these values. The idea is that there are recalibration tables local to each parallel task to minimize synchronization. The reduce merges the tables from different parallel tasks into the final, complete tables.

After the algorithm has computed the observations and errors for the recalibration tables, we derive the empirical quality score from these values. This is executed by a separate parallel algorithm (finalizeTable in Listing 2). It executes a parallel do loop that iterates over the entries of a table and calls a function that computes the empirical quality score from the observations and errors already recorded in the table. In contrast, the GATK 4 code updates the empirical quality score each time a table entry is created or updated. Calculating an empirical quality score requires computing a Bayesian estimation, which involves multiple floating point calculations and is therefore an expensive operation. Our strategy to only calculate the empirical quality score after all reads are processed produces the same result as the GATK 4 approach, while being computationally far more efficient.

Applying base quality score recalibration is defined as a filter in elPrep 4. Simplified pseudo code is shown in Listing 3. Since ApplyBQSR is an elPrep filter, it first defines and returns a function that operates on the header of a BAM file (first return). In this header function, we set up the tables (tab4 and tab5) that are derived from the recalibration tables that ApplyBQSR receives as input (tab1, tab2, tab3). This means that these tables are shared between the parallel tasks that are spawned for processing the reads. The header function also sets up a cache for storing recalibrated quality scores for specific types of bases.

The function that defines how to process a read (second return) shows how to update the base quality scores of a read. It first looks up the read group of the read, the sequencing cycle and context for each base. There is a loop that iterates over the bases of a read. For each base, it looks up the quality, cycle, and context to be able to look up the entries associated with this base in the recalibration tables (s1, s2, s3). First however, it attempts to see if this type of base was already recalibrated by looking it up in the cache. If an entry is found, the quality score for this base is immediately updated and this particular iteration is done (continue). Otherwise, if there is no cached result, it is calculated using a hierarchical Bayesian estimator (est). This base quality is thusly updated and the recalibrated quality score is saved in the cache. We observed a significant performance improvement by using such a caching mechanism for recalibrated quality scores in elPrep when compared to GATK 4, which has no such caching.

```
func BaseRecalibrator(reads, fasta, dbsnp) {
    ref := parseFasta(fasta)
    sites := parseVcf(dbsnp)
    tab1,tab2,tab3 := Table{}
    parallel.Reduce(
        reads,
        // map
        func lambda(read){
            if !recalibrateRead(read):
                return
            skip := read.computeSkip(sites)
            snps := read.computeSnps(ref)
            rg := read.getReadGroup()
            cycle := read.computeCycle()
            context := read.computeContext()
            for i := 0; i < len(read); i++ {
                if skip[i]:
                    continue
                qual := read.getQual(i)
                errors := snp[i]
                key1 := Key{rg, qual}
                tab1.update(key1, errors)
                key2 := Key{rg, qual, cycle[i]}
                tab2.update(key2, errors)
                key3 := Key{rg, qual, context[i]}
                tab3.update(key3, errors)
            }
        },
        // reduce
        func lambda (tab1,tab2,tab3,tab4,tab5,tab6) {
            tab1 = merge(tab1, tab4)
            tab2 = merge(tab2, tab5)
            tab3 = merge(tab3, tab6)
            return tab1, tab2, tab3
        }
    )
}

func finalizeTable(tab){
    parallel.Do {
        for key, entry in tab {
            entry.empiricalQual
                = entry.calcEmpiricalQual(key.qual)
        }
    }
}
```

Listing 2. Pseudo code for base recalibration in elPrep 4 (simplified).

```
func ApplyBQSR(tab1, tab2, tab3, lvl){
  return func lambda(header) {
      tab4 := initializeQuantizedQualityScores(tab1, lvl)
      tab5 := initializeCombinedBQSRTable(tab1)
      cache := Table{}
      return func lambda(read){
          rg := read.getReadGroup()
          cycle := read.computeCycle()
          context := read.computeContext()
          for i := 0; i < len(read); i++ {
              qual := read.getQual(i)
              key1 := Key{rg, qual}
              key2 := Key{rg, qual, cycle[i]}
              key3 := Key{rg, qual, context[i]}
              recalQual := cache[{key1,key2,key3}]
              if recalQual != nil:
                  read.Qual[i] = recalQual
                  continue
              s1 := tab1[rg,qual]
              s2 := tab2[rg,qual,cycle]
              s3 := tab3[rg,qual,context]
              prior := tab5[rg]
              est :=
                hierarchicalBayesianEstimate(prior,s1,s2,s3)
              recalQual = tab4[est]
              read.Qual[i] = recalQual
              cache[{key1,key2,key3}] = recalQual
              return true
              }
          }
      }
}
```

Listing 3. Pseudo code for applying base quality recalibration in elPrep 4 (simplified).

References

1. IEEE 1003.1-2017 - IEEE Approved Draft Standard for Information Technology - Portable Operating System Interface (POSIX(R)). IEEE; 2018.
